# Real-Time Fruit Recognition and Grasping Estimation for Robotic Apple Harvesting

**DOI:** 10.3390/s20195670

**Published:** 2020-10-04

**Authors:** Hanwen Kang, Hongyu Zhou, Xing Wang, Chao Chen

**Affiliations:** Laboratory of Motion Generation and Analysis, Faculty of Engineering, Monash University, Clayton, VIC 3800, Australia; hanwen.kang@monash.edu (H.K.); hugh.zhou@monash.edu (H.Z.); xing.wang2@monash.edu (X.W.)

**Keywords:** agricultural robot, deep learning, pointNet, autonomous harvesting, robotic harvesting, grasping estimation

## Abstract

Robotic harvesting shows a promising aspect in future development of agricultural industry. However, there are many challenges which are still presented in the development of a fully functional robotic harvesting system. Vision is one of the most important keys among these challenges. Traditional vision methods always suffer from defects in accuracy, robustness, and efficiency in real implementation environments. In this work, a fully deep learning-based vision method for autonomous apple harvesting is developed and evaluated. The developed method includes a light-weight one-stage detection and segmentation network for fruit recognition and a PointNet to process the point clouds and estimate a proper approach pose for each fruit before grasping. Fruit recognition network takes raw inputs from RGB-D camera and performs fruit detection and instance segmentation on RGB images. The PointNet grasping network combines depth information and results from the fruit recognition as input and outputs the approach pose of each fruit for robotic arm execution. The developed vision method is evaluated on RGB-D image data which are collected from both laboratory and orchard environments. Robotic harvesting experiments in both indoor and outdoor conditions are also included to validate the performance of the developed harvesting system. Experimental results show that the developed vision method can perform highly efficient and accurate to guide robotic harvesting. Overall, the developed robotic harvesting system achieves 0.8 on harvesting success rate and cycle time is 6.5 s.

## 1. Introduction

Robotic harvesting plays a significant role in the future development of the agricultural industry [1]. Vision is one of the key tasks among many challenges in the robotic harvesting [2]. There are environmental factors can affect the accuracy and robustness of the vision system, such as illumination and appearance variances, noisy background, and occlusion between objects [3]. Meanwhile, success rate of robotic harvesting in an unstructured environments can also be affected by the layout or distribution of the fruit within the workspace. To improve the success rate of robotic harvesting in such conditions, vision system should be capable of detaching crops from a proper pose [4,5]. Our previous work [6] developed a traditional grasping estimation method to perform harvesting. However, the performance of the traditional vision algorithms are always limited in complex and volatile environments. Inspired by the recent work of PointNet [7], this work proposes a fully deep neural network-based vision algorithm to perform real-time fruit recognition and grasping estimation for robotic apple harvesting. The proposed vision method includes two network models, a one-stage fruit recognition network and a PointNet-based grasping estimation network. The following contributions are highlighted in the paper:

- Proposing a computational-efficient light-weight one-stage instance segmentation network, Mobile-DasNet, to perform fruit detection and instance segmentation on sensory data.
- Proposing a modified PointNet-based network to perform fruit modelling and grasping estimation using point clouds from an RGB-D camera.
- Applying and combining the aforementioned two features into the design and build of the accurate robotic system towards autonomous fruit harvesting.

The rest of the paper is organised as follows. Section 2 reviews the related works on fruit recognition and grasping estimation. Section 3 introduces the methods of the proposed vision processing algorithm. The experimental setup and results are included in Section 4. In Section 5, conclusion and future works are presented.

## 2. Literature Review

### 2.1. Fruit Recognition

Fruit recognition is an essential task in the autonomous agricultural applications [8]. There are many methods which have been studied in decades, including the traditional method [9,10,11] and deep learning-based methods. Traditional methods apply hand-crafted features to encode the appearances of objects, and use machine-learning to perform detection or segmentation on such extracted features [12]. The performance of the traditional method is limited when a changing environment is presented [13]. By comparison, deep learning shows much better accuracy and robustness in such conditions [14]. Deep learning-based methods can be divided into two classes, two-stage detection and one-stage detection [15]. Two-stage detection divides the detection into region proposal and classification [16,17], while one-stage methods combines these two-steps [18,19]. Both two-stage and one-stage detection network have been widely studied in autonomous harvesting [20]. Bargoti and Underwood [21] applied Faster-RCNN to perform multi-classes fruit detection in orchard environments. Yu et al. [22] applied Mask-RCNN [23] to perform strawberry detection and instance segmentation in the non-structural environment. Liu et al. [24] developed a modified Faster-RCNN for kiwi fruit detection, which combined the information from RGB and NIR images and achieved accurate performance. Tian et al. [25] applied an improved Dense-YOLO to monitor apple growth in different stages. Koirala et al. [26] applied a light-weight YOLO-V2 model named as ’Mongo-YOLO’ to perform fruit load estimation. Kang et al. [27] introduced a novel multi-function neural network DasNet-v1 based on YOLO for real-time detection and semantic segmentation for both apples and branches in orchard environments. The detection and segmentation network with ResNet-101 backbone outperformed the corresponding task, while the network model with lightweight backbone also showed the best computation efficiency in the results.In the ensuing work [28], an enhanced deep neural network DasNet-v2 was developed, which achieved detection and instance segmentation on fruit and semantic segmentation on branches. The DasNet-v2 outperformed the previous neural network on the precision of apple detection and accuracy of semantic segmentation of branches and also applied instance segmentation on fruit as a new feature.

### 2.2. Grasping Estimation

Grasping estimation is one of the key challenges in the robotic grasp [29]. Grasping estimation methods can be divided into two categories: traditional methods and deep learning-based methods [30]. Traditional methods extract features or key points to estimate the object pose [31]. For the unknown objects, some assumptions have to be made, such as grasp the object along the principle axis [29]. The performance of the traditional methods is limited as noise or partial lose of point cloud can affect the accuracy and robustness of the estimation [32]. Some early deep learning-based methods recast the grasping estimation as an object detection task, which is to predict grasp pose from the 2D images [33]. Recently, with the development of the deep learning architecture for 3D point cloud processing [7,34], more studies focus on grasping estimation by using the 3D data, such as Grasp Pose Detection (GPD) [35] and PointNet GPD [36]. In the agricultural cases, most of works [37,38,39] pick fruit by translating towards the targets, which cannot secure the success rate of harvesting in unstructured environments. Lehnert et al. [40] applied a super-ellipsoid model to fit the sweep pepper and estimated the grasp pose by matching between the pre-defined shape and fruit. In their following work [41], they applied a utility function to find multiple candidate grasp poses during the harvesting, which can improve success rate but is not operational efficient. In this work, we combined latest development in both deep learning detection and grasp estimation, to demonstrate an accurate and robust vision method for fruit recognition and grasp estimation in a well-developed robotic harvesting system.

## 3. Methods and Materials

### 3.1. System Configuration

**Figure 1:**
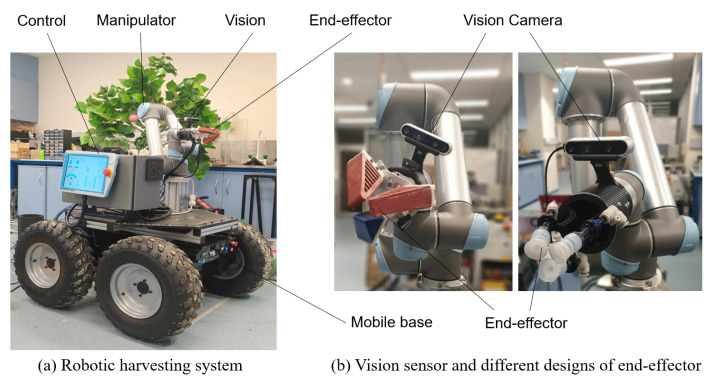
The developed robotic harvesting system that consists of mobile base, manipulator, vision camera, end-effector.

The developed robotic harvesting system includes a mobile moving vehicle base, an industrial robotic manipulator (Universal Robot UR5), a customised soft end-effector (includes a Intel D-435 RGBD vision camera), and a central control computer (DELL-INSPIRATION with an NVIDIA GTX-1070 GPU and Intel i7-6700 CPU), as shown in Figure 1. The control system is constructed based on Robot Operation System (ROS) in kinetic version [42] on the Linux Ubuntu 16.04. The communication between RGB-D camera, UR5 and computer is performed by RealSense communication package and universal-robot-ROS MoveIt! [43] with TackIK inverse kinematic solver [44].

The mobile base shown in Figure 1 is a customised moving vehicle, which mainly consists of a central control unit, four wheels with motor driven, 24 V power supply, and vehicle frames. The mobile base is designed to navigate to the desired location together with the whole robotic system. The universal robot (UR5) is an industrial standard robotic manipulator with 6 degree-of-freedoms. The manipulator helps perform the path planning together with the end-effector. Our end-effector adopted the design principle of the soft robotic grippers that have been explored significantly for robotic grasping application recently [45,46]. The proposed end-effector combines the compliant mechanism and the safe contact as a result of the fin-ray design and low elastic modulus material m4601, respectively. As for the vision subsystem, it mainly includes the RealSense RGB-D camera, which is used to capture the fruit images for further data processing. The processed data of fruit position and orientation will be used for the control of the robotic harvesting system.

**Figure 2:**
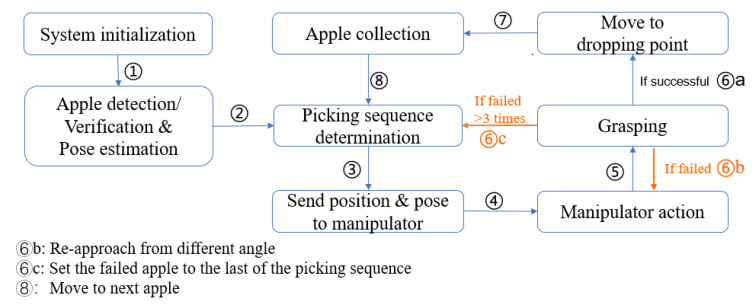
The working principle of the proposed apple harvesting robot.

The complete working process of the proposed robotic harvesting system is detailed in Figure 2.

#### Software Design

Our vision method includes two steps: fruit recognition and grasping estimation. In the first step, vision algorithm performs detection and instance segmentation on input RGB images. The predicted mask of each fruit is then combined with depth image to form the input point clouds of the PointNet. In the second step, the PointNet will predict the shape, size and approaching pose of each fruit by using the output from the first step. The methods of fruit detection and PointNet-based grasping estimation are presented in Section 3.2 and Section 3.3, respectively.

### 3.2. Fruit Recognition

#### 3.2.1. Network Architecture

**Figure 3:**
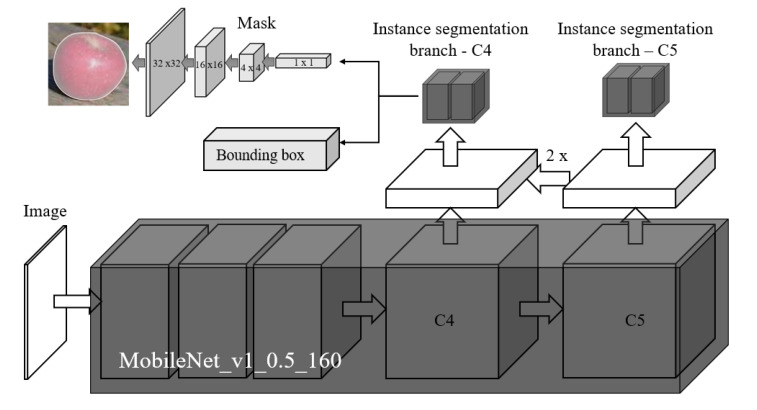
Network architecture of the Mobile-DasNet, which applies a light-weight backbone and a two-levels FPN to improve the computational efficiency.

An improved light-weight one-stage instance segmentation network ’Mobile-DasNet’ is developed in this research work, to perform fruit recognition, as shown in Figure 3. Compared to the previous network, DasNet [28], which applies resnet-50 [47] as the backbone and a three levels Feature Pyramid Network (FPN), the proposed Mobile-DasNet applies a light-weigth backbone ’MobileNet’ [48] and a two-levels FPN (receive feature maps from C4, and C5 levels) to improve its computational efficiency. The proposed Mobile-DasNet achieves a weight size of 20.5 MB and the average running speed of 63 FPS on an NVIDIA GTX-1070 GPU.

On each level of the FPN, an instance segmentation branch and an Atrous Spatial Pyramid Pooling (ASPP) block [49] is used. ASPP uses dilation convolution with different rates (e.g., 1, 2, 4) to process multiple-scale features. The instance segmentation branch includes two branches, mask segmentation branch and detection branch. Detection branch predicts a bounding box, confidence score, and class for a object within the grid. We use one preset anchor bounding box on each level of FPN, which are 50 × 50 and 120 × 120 on C4 and C5 levels, respectively. Binary mask segmentation branch follows the architecture design developed in Single Pixel Reconstruction Network (SPRNet) [50], which can predict a binary mask for objects from a single pixel within the feature maps. Mobile-DasNet also has a semantic segmentation branch for semantic segmentation of branch, which is not applied in this work.

#### 3.2.2. Network Training

There are 1200 images collected from different conditions to increase the diversity of the training data. For example, different time as day and night; different illumination as artificial lights, natural light, shadows, front lighting, side lighting and back lighting; different backgrounds as from the farms in Qingdao, China and Melbourne, Australia. These images are labelled by using LabelImage [51]. We use 600 images to train the network, 200 images as the validation set, and 400 images as the test set. Multiple image augmentations are introduced in training, including scaling (0.8–1.2), flip (horizontal only), rotation (±10°), and randomly adjustment on saturation (0.8–1.2) and brightness (0.8–1.2). Focal loss [52] and Adam-optimiser are used, and training resolution and batch size are 416 × 416 and 32, respectively. We first train network with learning rate 0.001 for 80 epochs and the train another 40 epochs with learning rate 0.0001.

### 3.3. Grasping Estimation

**Figure 4:**
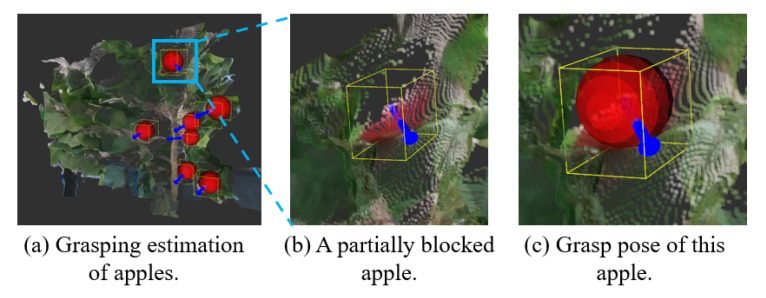
The proposed grasping estimation select vector from the fruit centre to surface centre of the visible part as grasp orientation.

An apple is modelled as a sphere in this work. In the natural environments, apples can be blocked from the view-angle of the RGB-D camera. Therefore, the visible part of the apple from the current view-angle of the RGB-D camera indicates the proper approaching pose for robotic arm to grasp target. We formulate the grasping estimation as an object pose estimation task, which is used in the Frustum PointNets [53]. We select vector from the geometric centre of the apple to visible surface centre of this apple from current view angle as approaching pose, as shown in Figure 4. Our method can take only 1-viewed point cloud as input and estimates the approaching pose, which significantly accelerate the operation speed.

#### 3.3.1. Pose Representation

**Figure 5:**
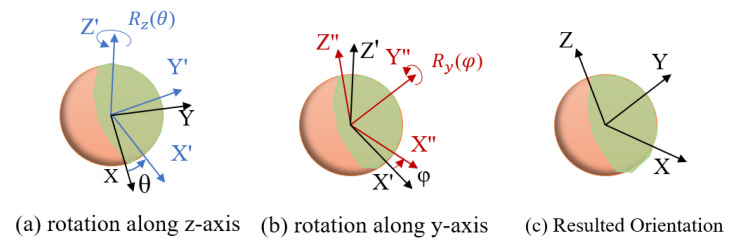
Euler-ZYX angle is applied to represent the orientation of the grasp pose.

The pose of an object in 3D space has 6 Degrees of Freedom (DoF), includes three positions (x, y, and z), and three rotations (θ, ϕ, and ψ, along Z-axis, Y-axis, and X-axis, respectively). We apply Euler-ZYX angle to represent the orientation of the grasp pose, as shown in Figure 5. The value of ψ is set to be zero as we assume that the fruit will not rotate along its stalk direction (X-axis). This assumption is made because apples are presented in a spherical shape. The grasp pose (GP) of an apple can be formulated as follow:

(1) $$T_{GP}=\left[\begin{array}{cccc} \cos\theta \cos\phi & -\sin\theta & \cos\theta \sin\phi & x\\ \sin\theta \cos\phi & \cos\theta & \sin\theta \sin\phi & y\\ -\sin\phi & 0 & \cos\phi & z\\ 0 & 0 & 0 & 1 \end{array}\right]$$

Therefore, a parameter list [x, y, z, θ, ϕ] is used to represent the grasp pose of the fruit.

#### 3.3.2. Pose Annotation

Grasping estimation block uses point clouds as the input and predicts the 3D Oriented Bounding Box (3D-OBB), oriented in grasp orientation, for each fruit. Each 3D-OBB includes six parameters, which are *x*, *y*, *z*, *r*, θ, ϕ. The position (*x*, *y*, *z*) represents the offsets on X-, Y-, Z-axis from the centre of point clouds to the centre of the apple, respectively. The parameter *r* represents the radius of the apple, as the apples is modelled as sphere. The length, width, and height can be derivated by radius. θ and ϕ represent the grasp pose of the fruit, as described in Section 3.3.1.

Since the values of the parameters *x*, *y*, *z*, and *r* may have large variances when dealing with prediction in different situations, a scale parameters *S* is introduced. We apply *S* to represent the mean scale (radius) of the apple, which equals 30 cm. The parameters *x*, *y*, *z*, and *r* are divided by *S* to obtain the united offset and radius ($x_{u}$, $y_{u}$, $z_{u}$, $r_{u}$). After remapping, the range of the $x_{u}$, $y_{u}$, $z_{u}$ is reduced to [−∞, ∞], and the range of $r_{u}$ are in [0, ∞]. To keep the grasp pose in the range of motion of the robotic arm, the θ and ϕ are limited in the range of [$-\frac{1}{4}\pi$, $\frac{1}{4}\pi$]. We divide the θ and ϕ by $\frac{1}{4}\pi$ to map the range of angle into the range of [−1,1]. The united θ and ϕ are denoted as $\theta_{u}$ and $\phi_{u}$. In total, we have six united parameters to predict the 3D-OBB for each fruit, which are [$x_{u}$, $y_{u}$, $z_{u}$, $r_{u}$, $\theta_{u}$, $\phi_{u}$]. Among these parameters, [$x_{u}$, $y_{u}$, $z_{u}$, $\theta_{u}$, $\phi_{u}$] represent the grasp pose of the fruit, $r_{u}$ controls the shape of 3D-OBB.

**Figure 6:**
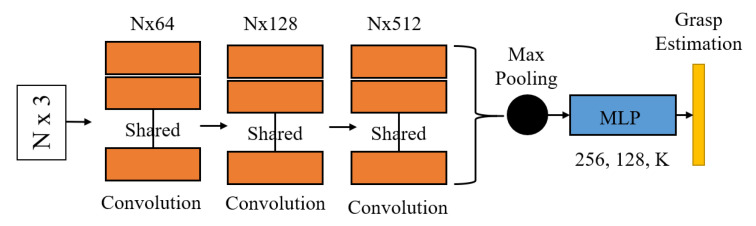
Network architecture of the PointNet applied in grasping estimation.

PointNet [7] is a deep neural network architecture which can perform classification, segmentation, or other tasks on point clouds. PointNet uses raw point clouds of the object as input and does not require any pre-processing. The architecture of the PointNet is shown in Figure 6. PointNet uses an n × 3 (n is the number of points) unordered point clouds as input. Firstly, PointNet applies convolution operations to extract a multiple dimensional feature vector on each point. Then, a symmetric function is used to extract the features of the point clouds on each dimension of the feature vector.

(2) $$f\left(x_{1},\;x_{2},\;\ldots ,\;x_{n}\right)=g\left(h\left(x_{1}\right),\;h\left(x_{2}\right),\;\ldots ,\;h\left(x_{n}\right)\right)$$

where *g* is a symmetric function and *f* is the extracted features from the set. PointNet applies max-pooling as the symmetric function. So that it can learn numbers of features from point set and invariant to input permutation. The generated feature vectors are further processed by Multi-Layer Perception (MLP) (fully connected layer in PointNet), to perform classification of the input point clouds.

#### 3.3.3. PointNet Architecture

In this work, PointNet predicts six parameters [$x_{u}$, $y_{u}$, $z_{u}$, $r_{u}$, $\theta_{u}$, $\phi_{u}$]. The range of the parameters $x_{u}$, $y_{u}$, and $z_{u}$ are in [−∞, ∞], hence we do not applies an activation function on these three parameters. The range of the $r_{u}$ are from 0 to ∞, the exponential function is used as activation. The range of the $\theta_{u}$, $\phi_{u}$ are from −1 to 1, hence a tanh activation function is applied. The PointNet output before activation are denoted as [$x_{p}$, $y_{p}$, $z_{p}$, $r_{p}$, $\theta_{p}$, $\phi_{p}$]. Therefore, we have

(3) $$\left[\begin{array}{c} x_{u}\\ y_{u}\\ z_{u} \end{array}\right]=\left[\begin{array}{c} x_{p}\\ y_{p}\\ z_{p} \end{array}\right],\left[\begin{array}{c} r_{u}\\ \theta_{u}\\ \phi_{u} \end{array}\right]=\left[\begin{array}{c} \exp(r_{p})\\ \tanh(\theta_{p})\\ \tanh(\phi_{p}) \end{array}\right]$$

The output of the PointNet can be mapped back to their original value by following the description in Section 3.3.2.

#### 3.3.4. Network Training

The data labelling is performed on our customised labelling tool. We collect 570 samples (320 in lab, 250 in orchards). We use 300 samples as training set, 50 samples as validation set, and 220 samples as test set. Scaling (0.8 to 1.2), translation (−15 cm to 15 cm), rotation (−10° to 10° on θ and ϕ), Gaussian noise, and outliers are used in training. The squared error is used as the training loss. The learning rate and decay rate are 0.0001, 0.6/epoch, respectively. We train the network for 100 epochs with batch size equals 64.

## 4. Experiment and Discussion

### 4.1. Experiment Setup

The developed vision algorithm was evaluated using both image data and the robotic harvesting experiment in indoor and outdoor environments. We used an Intel RGB-D camera on the robotic arm to detect and locate the spatial location of apples (in instance masks in 2D images or 3D point clouds). As the RGB-D camera has been fixed on the robotic arm, we can map the detected objects from the RGB-D camera coordinate to the robotic arm coordinate. In this way, we obtain the position of the target in agriculture robot coordinate. The distance between robotic arm and apples was measured by using the RGB-D camera. The Grasping module only estimates the centre and grasping pose from the obtained 3D point clouds, to accurately guide the robotic harvesting. In the first experiment, we tested the developed method on 110 images respectively in the laboratory environment and orchard environment. In the robotic harvesting experiment, we applied the developed harvesting system to perform the apple harvesting on a real apple trees in both lab and outdoor environments. We applied IoU to evaluate the accuracy of 3D localisation and shape estimation of the fruit. 3D Axis Aligned Bounding Boxes (3D-AABB) was used to simplify the IoU calculation of 3D bounding box [54], which was denoted as IoU${}_{3D}$ in this paper. We set 0.75 (thres${}_{IoU}$) as the threshold value for IoU${}_{3D}$ to determine the accuracy of fruit shape prediction. In terms of the evaluation of grasping estimation, we applied Mean Squared Error (MSE) between the predicted value and ground truth value of approaching pose, as it can intuitively show the accuracy of predicted results.

### 4.2. Image Data Experiments

In this experiment, we compared the developed deep learning-based method with other two traditional methods, which were sphere Random Sample Consensus (sphere-RANSAC) [55] and sphere Hough Transform (sphere-HT) [56]. Both RANSAC and HT algorithms took point clouds as input and generated the prediction of the fruit shape. This comparison was conducted on RGB-D images collected from both laboratory and orchard environments. In the experiment, we also included condition of dense clutter, to evaluate the performance of algorithm when fruit are close to each other.

#### 4.2.1. Experiments in Laboratory Environment

**Figure 7:**
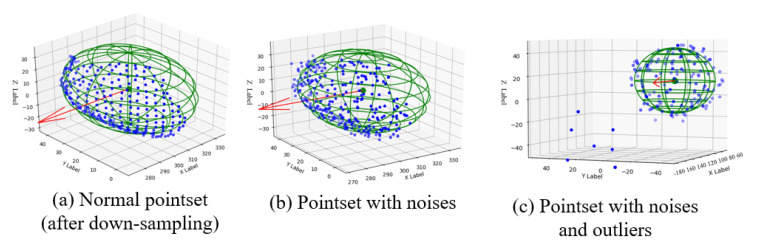
Pointset under different conditions, green sphere is the ground truth of the fruit shape.

The experimental results of different methods in several conditions are shown in Table 1. Experimental results showed that PointNet-based method significantly increases the localisation accuracy (0.94 in normal condition) of the 3D bounding box, which was much higher the accuracy of the RANSAC and HT algorithms (0.82 and 0.81 in normal conditions, respectively). To evaluate the robustness of different methods when dealing with noisy and outlier conditions, we artificially added Gaussian noise (mean equals 0, variance equals 2cm) and outlier (1% to 5% in the total number of point clouds) into the point clouds, which are shown in Figure 7. Three methods achieved similar performance on robustness when dealing with outliers condition. Both RANSAC and HT applied vote framework to estimate the primitives of the shape, which was robust to the outlier. However, PointNet-based methods showed much better robustness when dealing with noisy data, which only showed a 3% drop on results from the normal condition, while both RANSAC and HT showed significant decrease of accuracy compared to the PointNet. In the dense clutter case, PointNet showed better accuracy compared to other two methods. Experimental results suggested that PointNet-based method improves accuracy and robustness of grasping estimation compared to the traditional methods.

In the evaluation of approaching pose prediction, PointNet-based method also showed accurate performance in the experimental results, as shown in Table 2. The MSE between predicted grasp pose and ground truth grasp pose was 4.2°. Experimental results showed that PointNet grasping estimation can accurately and robustly determine the grasp orientation of the objects in noisy, outlier presented, and dense clutter conditions.

#### 4.2.2. Experiments in Orchards Environment

**Figure 8:**
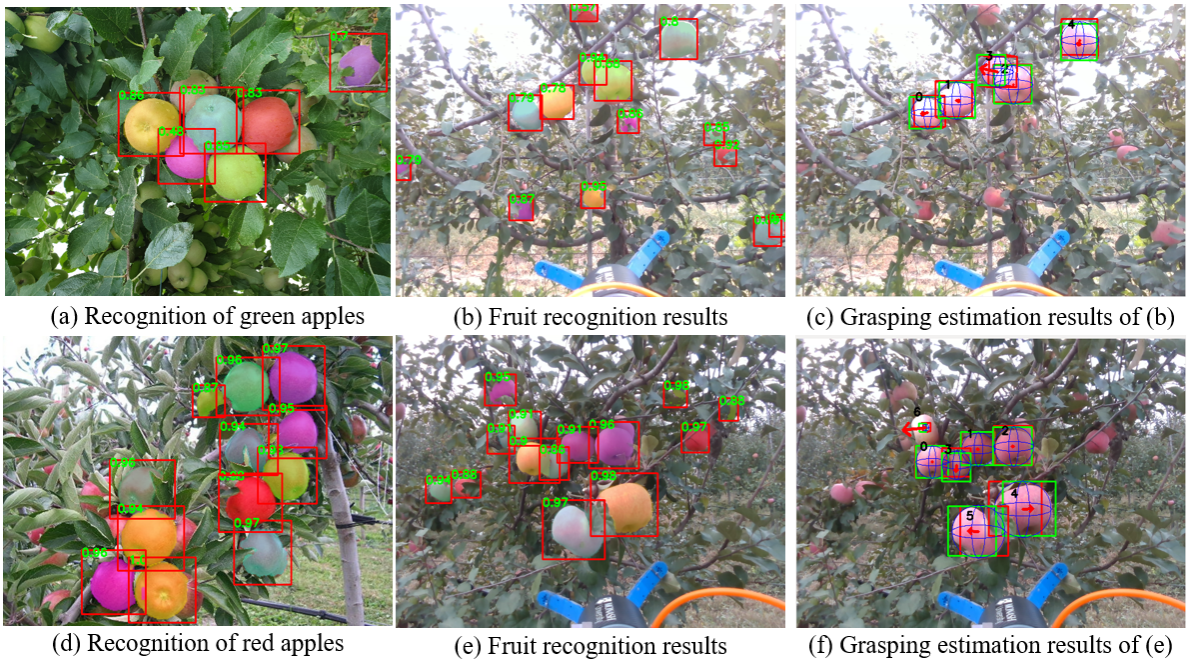
Fruit recognition and grasping estimation experiments in orchard scenario.

In this experiment, we performed the fruit recognition and PointNet grasping estimation on the collected RGB-D images from apple orchards. F${}_{1}$ score and IoU were used as the evaluation metric on fruit detection and segmentation, respectively. Table 3 and Table 4 showed the performance of the DasNet/Mobile-DasNet and PointNet grasping estimation. It can be seen this Mobile-DasNet achieves much faster running speed compared with DasNet [28], with a value of 63 FPS compared to 25 FPS. Experimental results showed that both DasNet and Mobile-DasNet can perform well on fruit recognition in orchard environment (as shown in Figure 8).

Table 4 showed the performance comparison between PointNet grasping estimation, RANSAC, and HT. In the orchard environments, grasping estimation was more challenging compared to the indoor environments. In this condition, the performance of the RANSAC and HT showed the significant decrease from the indoor experiment while PointNet grasping estimation showed better robustness. The IoU${}_{3D}$ achieved by PointNet grasping estimation, RANSAC, and HT in orchard scenario were 0.88, 0.76, and 0.78, respectively. In terms of the grasp orientation estimation, PointNet grasping estimations showed robust performance in dealing with flawed sensory data. The mean error of orientation estimation by using PointNet grasping estimation was 6.6°, which was still within the accepted range of orientation error. The experimental results of grasping estimation by using PointNet grasping estimation in orchard scenario are shown in Figure 8.

### 4.3. Experiments of Robotic Harvesting

**Figure 9:**
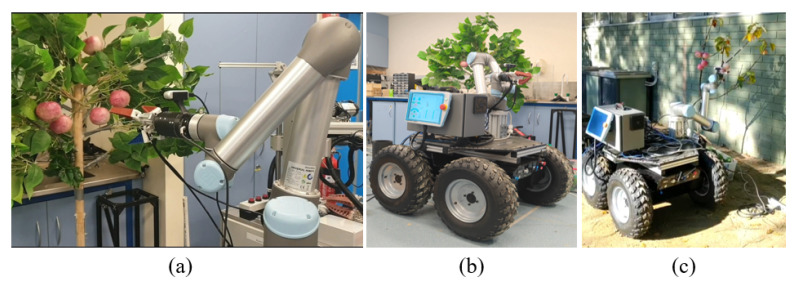
Experiment setup in (**a**,**b**) indoor laboratory and (**c**) outdoor environments.

**Figure 10:**
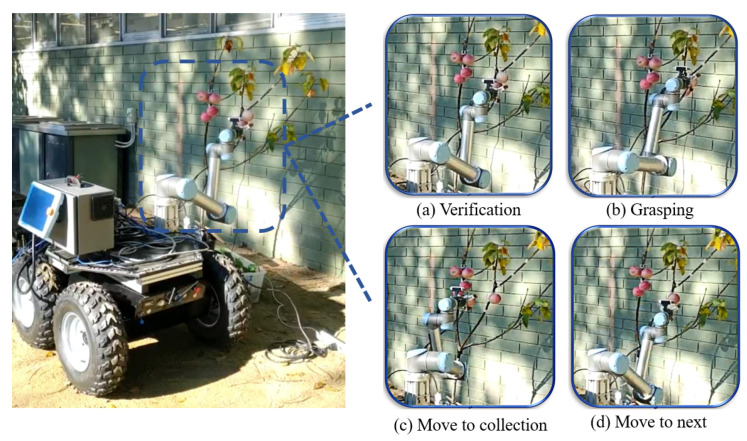
The process for robotic harvesting experiment in outdoor environment.

The developed robotic harvesting system was validated in both indoor laboratory and outdoor environments, which was shown in Figure 9. We randomly arranged number, distribution, and location of apples on the apple tree to evaluate the success rate of the robotic harvesting. The robotic grasping included four steps: sensing, verification, grasping, and collection, as shown in Figure 10. We tested and compared two different harvesting strategies, which were the naive harvesting method and Pose prediction enabled harvesting method, as shown in Table 5. Naive harvesting method only translated to detach fruit while not considering the grasping pose of each fruit.

From the experimental results in Table 5, the accuracy of grasping pose estimation was lower than the performance achieved on the RGB-D image data, in both indoor and outdoor conditions. We found that this performance reduction was due to the fluctuation of end-effector during the robotic arm moving, which may generate flawed sensory data. Therefore, we added 0.5 s delay after each motion of robotic arm to ensure the quality of input sensory data. There were several reasons leading to unsuccessful grasping, which included loose grasp and dense clutter. In the first conditions, our customised three-fingers end-effector may lose contact with target fruit with one or two fingers (contacting with nearby branches instead or receiving not accurate grasping pose), which can cause the target to slip off from the gripper and lead to the failure, while under dense clutter conditions, the gripper can easily touch adjacent fruit and cause these neighbour fruit to drop. Pose prediction enabled harvesting significantly increased the success rate of robotic harvesting and reduced the re-attempt times in both indoor and outdoor environments, compared to the naive harvesting method. The cycle time of each attempts for naive harvesting method and Pose prediction enabled harvesting method was 4 s and 6.5 s, respectively. Overall, our developed vision method showed a promising performance in improving the accuracy and robustness of robotic harvesting system, which was validated in both indoor and outdoor environments.

### 4.4. Discussion

**Figure 11:**
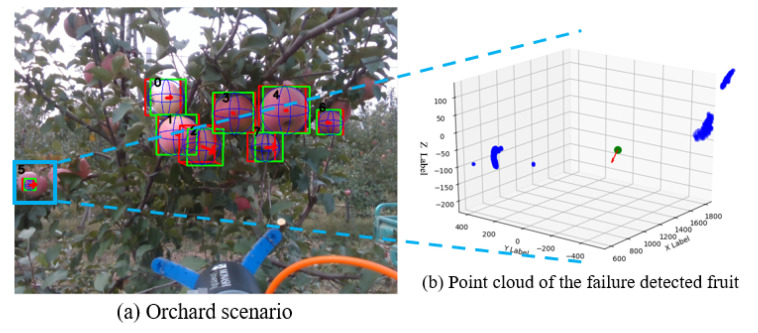
Failure grasping estimation in orchard scenario.

In the image data experiments, the comparison between the proposed deep learning-based algorithm, PointNet, and the traditional algorithms such as RANSAC and HT, indicated that the proposed PointNet demonstrated much superior robustness when processing the data with noise. This difference is because the noise will influence the accuracy of vote framework to a large extent. Our method also showed the best accuracy when identifying the fruit shape estimation in dense clutter condition among all three methods. Besides, the experiment results indicated that PointNet predicted the approaching pose while grasping accurately and robustly in the complex conditions with noise, outlier and dense clutter. The experiment validated that both DasNet and Mobile-DasNet can perform well on fruit recognition and instance segmentation in orchard environments. The proposed one-stage detector for fruit recognition shows its accuracy and computational efficiency. This light-weight Mobile-DasNet achieved 0.851, 0.826, 0.9 on F1 score, recall and accuracy on fruit detection and an accuracy of 0.82 on instance segmentation. With this one-stage detector, the detection and segmentation tasks of the fruit are accelerated, which shortens the average cycle time for fruit harvesting. As for the possible improvement of the proposed methods, the function of proposed PointNet and Mobile-DasNet can be potentially combined into one stage. With the fruit detection, segmentation together with fruit modelling, grasping estimation achieved in one stage, the real time performance of the robotic harvesting system is expected to be improved. The major reason leading to the failed estimation of grasping pose was the defect of sensory data, as shown in Figure 11, which came from the test data set. While for the apple highlighted in blue boundary box in Figure 11a, as the generation of its point cloud failed in the first place, as shown in Figure 11b, the grasping estimation did not proceed and was treated as a failure grasping estimation. In this case, there was not an ideal value in the grasping estimation as there was not ground truth. In this condition, PointNet grasping estimation will always tend to predict a sphere with small value of radius, which can be easily filtered as outliers during the implementation.

As for the robotic harvesting, our proposed harvesting method outperformed the naive harvesting method not only in the higher harvesting success rate, but also in the reduced re-attempt times, while the former method was enabled by pose estimation and the latter can only translate to the detected fruit. There were several reasons leading to unsuccessful grasping, which included loose grasp and dense clutter. In the first conditions, our customised three-fingers end-effector may lose contact with target fruit with one or two fingers (contacting with nearby branches instead or receiving not accurate grasping pose), which can cause the target to slip off from the gripper and lead to failure. Under dense clutter conditions, the gripper can easily touch adjacent fruit and cause these neighbour fruit to drop. Pose prediction enabled harvesting significantly increased the success rate of robotic harvesting and reduced the re-attempt times in both indoor and outdoor environments, compared to the naive harvesting method.

## 5. Conclusions and Future Work

In this work, a fully deep learning neural network-based fruit recognition and grasping estimation method was proposed and experimentally validated. The proposed method included a multi-functional network that can perform fruit detection and instance segmentation at one-stage, and a PointNet neural network to process the point cloud of the fruit and grasping estimation to determine the proper grasp pose for each fruit. This grasping pose is important when performing autonomous fruit harvesting. The proposed multi-function fruit recognition network and PointNet grasping estimation network were trained and validated on RGB-D images taken from both laboratory and orchard environments. Experimental results showed that the proposed method could accurately perform visual perception and grasping estimation. The proposed Mobile-DasNet achieved 0.851, 0.826, 0.9 on F1 score, recall and accuracy on fruit detection and an accuracy of 0.82 on instance segmentation. As for the grasping estimation. The IoU3D achieved by PointNet grasping estimation, RANSAC, and HT algorithms in orchard scenario were 0.88, 0.76, and 0.78, respectively. It can be seen that the PointNet outperformed the other two traditional algorithms. Our developed robotic harvesting system was also tested in the indoor and outdoor environments, which showed promising performance in both accuracy, robustness, and operational speed. Overall, the developed robotic harvesting system achieves 0.8 on harvesting success rate and cycle time is 6.5 s. In the future, we will further optimise the vision algorithm in terms of accuracy, robustness, and speed. Moreover, the soft robotic finger based end-effector can be further optimised to improve its success rate and efficiency of grasping under different conditions.

## Figures and Tables

**Table 1:** Accuracy of the fruit shape estimation by using PointNet, RANSAC, and HT in different tests.

	Normal	Noise	Outlier	Dense Clutter	Noise+Outlier+Dense Clutter
PointNet	0.94	0.92	0.93	0.91	0.89
RANSAC	0.82	0.71	0.81	0.74	0.61
HT	0.81	0.67	0.79	0.73	0.63

**Table 2:** Mean error of grasp orientation estimation by using PointNet in different tests.

	Normal	Noise	Outlier	Dense Clutter	Noise+Outlier+Dense Clutter
PointNet	4.2°	5.4°	4.6°	6.8°	7.5°

**Table 3:** Performance of fruit recognition in orchard environments.

	F${}_{1}$ Score	mAP${}_{50}$	Recall	Accuracy	IoU${}_{mask}$	Running Speed
DasNet	0.884	0.905	0.88	0.91	0.873	25 FPS
Mobile-DasNet	0.851	0.863	0.826	0.9	0.82	63 FPS

**Table 4:** Evaluation on grasping estimation by using PointNet in different tests in the orchard scenario.

	PointNet	RANSAC	HT
Accuracy	0.88	0.76	0.78
Grasp Orientation	6.6°	-	-

**Table 5:** Experimental results on robotic grasp by using PointNet grasping estimation in Laboratory scenario.

	Harvesting Method	Pose Prediction Success Rate	Harvesting Success Rate	Re-Attempt Times
Indoor	Naive	-	0.73	1.5
Indoor	Pose prediction enabled	0.88	0.85	1.2
Outdoor	Naive	-	0.72	1.6
Outdoor	Pose prediction enabled	0.83	0.8	1.3
